# Alterations in microRNA Expression during Hematopoietic Stem Cell Mobilization

**DOI:** 10.3390/biology10070668

**Published:** 2021-07-15

**Authors:** Mateusz Nowicki, Janusz Szemraj, Agnieszka Wierzbowska, Agnieszka Pluta, Olga Grzybowska-Izydorczyk, Aleksandra Nowicka, Piotr Stelmach, Magdalena Czemerska, Anna Szmigielska-Kapłon

**Affiliations:** 1 Department of Hematology, Copernicus Memorial Hospital in Lodz Comprehensive Cancer Center and Traumatology, 93-513 Łódź, Poland; agnieszka.wierzbowska@umed.lodz.pl (A.W.); agnieszka.pluta@umed.lodz.pl (A.P.); olga.grzybowska-izydorczyk@umed.lodz.pl (O.G.-I.); piotr.stelmach@umed.lodz.pl (P.S.); magdalena.czemerska@umed.lodz.pl (M.C.); anna.szmigielska-kaplon@umed.lodz.pl (A.S.-K.); 2 Department of Medical Biochemistry, Medical University of Lodz, 92-215 Łódź, Poland; janusz.szemraj@umed.lodz.pl (J.S.); aleksandranowicka9003@gmail.com (A.N.); 3 Department of Hematology, Medical University of Lodz, 93-510 Łódź, Poland

**Keywords:** miRNA, hsa-miR-146a-5p, mobilization, CD34+, multiple myeloma, hematopoietic stem cells

## Abstract

**Simple Summary:**

Lymphoproliferative disorders comprise a heterogeneous group of hematological malignancies characterized by abnormal lymphocyte proliferation. Autologous hematopoietic stem cell transplantation plays a very important role in the treatment of lymphoproliferative diseases. The key element in this process is the effective mobilization of hematopoietic cells from the marrow niche to the peripheral blood. Mobilization of HSC is regulated by many factors, out of which miRNAs present in the hematopoietic niche via targeting cytokines, and signaling pathways may play an important regulatory role. This study investigated the expression of selected miRNAs in patients with multiple myeloma, Hodgkin’s lymphomas, and non-Hodgkin’s lymphomas undergoing mobilization procedures. The aim of the study was to evaluate the expression of hsa-miR-15a-5p, hsa-miR-16-5p, hsa-miR-34a-5p, hsa-miR-126-3p, hsa-miR-146a-5p, hsa-miR-155-5p, and hsa-miR-223-3p during the mobilization procedure, and to assess their role in mobilization efficacy. The level of miRNAs was tested at two time points before the initiation of mobilization and on the day of the first apheresis. Our results suggest that the investigated miRNAs, especially hsa-miR-146a-5p, may influence the efficacy of HSC mobilization.

**Abstract:**

microRNAs play an important role in the regulation of gene expression, cell fate, hematopoiesis, and may influence the efficacy of CD34+ cell mobilization. The present study examines the role of hsa-miR-15a-5p, hsa-miR-16-5p, hsa-miR-34a-5p, hsa-miR-126-3p, hsa-miR-146a-5p, hsa-miR-155-5p, and hsa-miR-223-3p in the course of hematopoietic stem cell mobilization. The numbers of CD34+ cells collected in patients with hematological malignancies (39 multiple myelomas, 11 lymphomas) were determined during mobilization for an autologous hematopoietic stem cell transplantation. The miRNA level was evaluated by RT-PCR. Compared to baseline, a significant decline in hsa-miR-15a-5p, hsa-miR-16-5p, hsa-miR-126-3p, hsa-miR-146a-5p, and hsa-miR-155-5p was observed on the day of the first apheresis (day A). An increase was observed only in the expression of hsa-miR-34a-5p. On day A, a negative correlation was found between hsa-miR-15a-5p and hsa-miR-146a-5p levels and the number of CD34+ cells in peripheral blood. A negative correlation was observed between hsa-miR-146a-5p and the number of collected CD34+ cells after the first apheresis. Good mobilizers, defined according to GITMO criteria, demonstrated a lower hsa-miR-146a-5p level on day A than poor mobilizers. Patients from the hsa-miR-146a-5p “low expressors” collected more CD34+ cells than “high expressors”. Our results suggest that the investigated miRNAs, especially hsa-miR-146a-5p, may influence the efficacy of HSC mobilization.

## 1. Introduction

Lymphoproliferative disorders (LD) comprise a heterogeneous group of hematological diseases that demonstrate a dysregulated proliferation of lymphoid lineage cells [1]. An important role in LD treatment is played by high-dose chemotherapy with autologous hematopoietic stem cell transplantation (auto-HSCT). Successful mobilization of hematopoietic stem cells (HSC) is a crucial step in this procedure. In the mobilization process, mononuclear cells with the CD34+ antigen are released from the bone marrow after chemotherapy and the administration of granulocyte colony-stimulating factor (G-CSF) [2]. Mobilization of HSC and their homing after auto-HSCT are regulated by many factors, among which cytokines present in the hematopoietic niche play an important regulatory role [3]. Changes in their expression affect the ligand–receptor system, significantly influencing the signal pathways active in hematopoiesis [3,4]. CD34+ cell migration is also significantly influenced by microRNAs (miRNAs). miRNAs are small, endogenous RNA molecules (consisting of 19–25 nucleotides) that bind to the 3′ or 5′ UTR region of the messenger RNA (mRNA) and usually cause mRNA degradation and translation inhibition. It has been reported that miRNAs can also activate translation or regulate transcription. miRNAs influence the regulation of gene expression in physiological and pathological conditions [5,6]. miRNA molecules are known to significantly affect the expression of genes responsible for the angiogenesis, apoptosis, development, and differentiation of HSC [5,7].

An extremely important step in the treatment of multiple myeloma and lymphomas is the adequate mobilization of HSC, as this is essential for obtaining a sufficient number of CD34+ cells needed for auto-HSCT. The parameters influencing the efficacy of mobilization include the clinical condition of the patient, the chemotherapy regimen, and the duration of G-CSF administration [8,9,10]. Although such chemotherapy and G-CSF mobilization protocols have been performed for many years, the exact mechanism by which they act remains unclear. 

**Figure 1:**
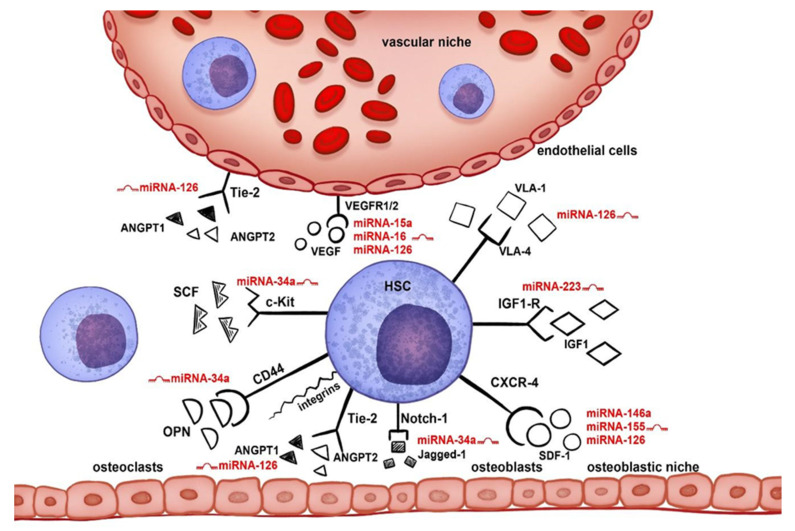
Influence of miRNAs on individual signaling pathways in the marrow niche. The following ligand–receptor interactions play a key role in the HSC migration in the bone marrow niche: CXCL12 (SDF-1)/CXCR4, NOTCH1/JAG1 (Jagged-1), ANGPT1/ANGPT2/TEK (Tie-2), SPP1 (OPN)/CD44/integrin receptors, KITLG (SCF)/KIT (c-Kit), VEGFA (VEGF)/FLT1/KDR (VEGFR1/2), VCAM1/ITGA4 (VLA-4), and IGF1/IGF1R, which are significantly influenced by the selected miRNAs: hsa-miR-15a-5p, hsa-miR-16-5p, hsa-miR-126-3p, hsa-miR-146a-5p, hsa-miR-223-3p, hsa-miR-34a-5p, and hsa-miR-155-5p.

HSC migration is primarily controlled by several key factors associated with the bone marrow niche, which affects crucial aspects of mobilization: duration of apheresis, the number of apheresis, and acquisition of the optimal number of CD34+ cells for auto-HSCT. Recent research indicates that hsa-miR-15a-5p, hsa-miR-16-5p, hsa-miR-34a-5p, hsa-miR-126-3p, hsa-miR-146a-5p, and hsa-miR-155-5p play an important regulatory role in the hematopoietic niche and significantly affect HSC migration [11,12,13,14,15,16,17,18,19,20,21,22,23,24] (Figure 1). The expression of the abovementioned miRNAs was the subject of our research in the course of the auto-HSCT [25]. In this paper, we evaluate their roles in HSC migration during mobilization.

Alterations in the expression of hsa-miR-15a-5p and hsa-miR-16-5p are commonly observed in solid tumors, chronic lymphocytic leukemia, lymphomas, and in more than half of multiple myeloma patients [26,27]. In follicular lymphoma, hsa-miR-16p downregulates the expression of BCL2 and, hence, acts pro-apoptotic [28]. The hsa-miR-15a-5p/hsa-miR-16-5p cluster downregulates the expression of vascular endothelial growth factor A (VEGFA) and influences angiogenesis and regeneration after auto-HSCT. A negative correlation was observed between hsa-miR-15a-5p/hsa-miR-16-5p expression and the VEGFA level in myeloma cells [13]. Alterations in the expression of hsa-miR-15a-5p/hsa-miR-16-5p are associated with chemoresistance [29]. hsa-miR-15a-5p/hsa-miR-16-5p inhibits the serine/threonine kinase 1 (AKT1) signaling pathway, which is responsible for adhesion and migration in B cells [30]. Decreased expression of these miRNAs inhibits apoptosis, promotes angiogenesis, and encourages the proliferation of tumor cells [29,31].

Hsa-miR-34a-5p is considered to be a tumor suppressor, and its increased expression in multiple myeloma stem cells is associated with decreased cell proliferation and reduced tumor growth. [21,32,33]. Together with hsa-miR-155-5p, hsa-miR-34a-5p is involved in the pathogenesis of lymphoma [34]. Increased expression of hsa-miR-34a-5p upregulates the level of Diablo IAP-binding mitochondrial protein (DIABLO), resulting in the inhibition of myeloma cell growth and altered sensitivity of cancer cells to chemotherapy [33]. Hsa-miR-34a-5p inhibits cell viability by inactivating the AKT1 and mitogen-activated protein kinase 1/3 (MAPK1/MAPK3) pathways, which are both involved in the regulation of HSC motility, proliferation, and survival [21,35].

Hsa-miR-126-3p is expressed by HSC, megakaryocytes, and endothelial cells (EC) [36,37]. Moreover, the elevated expression of hsa-miR-126-3p is observed in G-CSF-mobilized CD34+ cells [36]. Hsa-miR-126-3p regulates the migration of hematopoietic and progenitor stem cells (HPSC) by targeting the vascular cell adhesion molecule 1 (VCAM1) [38]. G-CSF stimulation promotes the accumulation of microvesicles containing hsa-miR-126-3p at the time of mobilization treatment; this is associated with the downregulation of VCAM1 expression on the bone marrow cell surface [15,38]. The resulting low level of VCAM1 improves the release of HPSC from the bone marrow niche during mobilization and impairs homing after HSCT [15,36,38]. Hsa-miR-126-3p influences HSC proliferation, survival, and migration by affecting the phosphatidylinositol-4,5-bisphosphate 3-kinase catalytic subunit alpha serine/threonine kinase 1 (PIK3CA/AKT1) signaling axis [39].

Hsa-miR-146a-5p is expressed on HSCs and affects the bone marrow niche homeostasis [40,41]. This prevents an excessive inflammatory response by regulating the nuclear factor kappa B subunit 1 (NFKB1) pathway and inhibiting the expression of the TNF receptor-associated factor 6 (TRAF6) and interleukin 1 receptor-associated kinase 1 (IRAK1) genes [42]. In addition, hsa-miR-146a-5p affects the growth of myeloid and lymphoid tumors. This significantly influences the mobilization of hematopoietic cells, as well as the regeneration after auto-HSCT [43,44]. During G-CSF administration, hsa-miR-146a-5p interferes with the C-X-C motif chemokine ligand 12/C-X-C motif chemokine receptor 4 (CXCL12/CXCR4) signaling axis, resulting in more efficient HSC mobilization and slower homing after auto-HSCT [20].

Hsa-miR-155-5p affects the development of myeloproliferative diseases, and its expression is also increased in patients with lymphoma [45,46,47,48,49]. In the indolent B-cell non-Hodgkin lymphomas, hsa-miR-155-5p via targeting SPI1 mRNA leads to the inhibition of B-cell differentiation [28]. In myeloma patients, fluctuations in the expression of hsa-miR-155-5p may be a prognostic factor in the course of the disease [50]. Furthermore, increased hsa-miR-155-5p expression reduces proteasome activity and increases myeloma cell sensitivity to bortezomib [51]. An increased expression of hsa-miR-155-5p was observed in CD34+ progenitor cells in G-CSF mobilized patients [36]. Hsa-miR-155-5p affects the CXCL12/CXCR4 signaling axis via AKT1 activation, and influences the effectiveness of mobilization [22,23]. The expression of hsa-miR-155-5p is associated with HSC differentiation [47]. Elevated hsa-miR-155-5p levels inhibit the PIK3CA/AKT1 signaling pathway, which promotes cell proliferation and inhibits apoptosis [34,52,53].

In hematological malignancies, hsa-miR-223-3p is a tumor-suppressive molecule that plays a significant role in cancer development [54,55]. Its abnormal expression is observed in B-cell malignancies, MM, and acute myeloid leukemia (AML) [56]. Downregulation of NOTCH1 signaling in mesenchymal stem cells from multiple myeloma patients leads to an elevated expression of hsa-miR-223-3p and a decrease in the VEGFA level [32]. Hsa-miR-223-3p is associated with myeloid lineage development by promoting granulopoiesis while repressing macrophage differentiation [57]. On the other hand, hsa-miR-223-3p is an important molecule participating in the differentiation and maturation of hematopoietic progenitor cells (HPC) [58]. It is also essential for maintaining the homeostasis of mature neutrophils and limiting inflammation [55,58]. Hsa-miR-223-3p is involved in the development and maturation of myeloid progenitors to granulocytic, erythroid, monocyte/macrophage lines [56,58]. Hsa-miR-223-3p regulates the PIK3CA/AKT1 axis and controls cell survival, proliferation, and migration by targeting the insulin-like growth factor 1 receptor (IGF1R) [55,59]. An elevated expression of hsa-miR-223-3p was observed in CD34+ peripheral blood stem cells (PBSC) [23]. The downregulation of hsa-miR-223-3p impairs granulopoiesis and progenitor cell differentiation [55,60,61].

The hematopoietic niche forms a unique microenvironment for HSC development that is modulated by a complicated network of molecules, particularly key regulators: miRNAs. Migration of the HSC during mobilization is therefore influenced by a variety of factors, including competing ones.

This study evaluated the expression of selected miRNAs during the mobilization procedure and assessed their role in mobilization efficacy.

## 2. Materials and Methods

Twenty-five females and twenty-five males with a median age of 60 years were enrolled in the study. The investigated cohort consisted of thirty-nine multiple myeloma (MM), seven non-Hodgkin lymphoma (NHL), and four Hodgkin lymphoma (HL) patients. More comprehensive clinical data are presented in Table 1. The blood plasma samples were collected at two time points: before hematopoietic stem cell mobilization chemotherapy (day 0) and on the day of the first apheresis (day A).

The blood was centrifuged at 1000× *g* for 10 min at 4 °C. Plasma samples were stored frozen at −80 °C. MiRNA expression was assessed in peripheral blood (PB).

The mobilization regimens consisted of Endoxan (cyclophosphamide), Ara-C (cytarabine), DCEP (Dexamethasone, Cyclophosphamide, Cisplatin, Etoposide), plus G-CSF or G-CSF in monotherapy for patients with MM. For patients with lymphoma, the mobilization chemotherapy consisted of ICE (Ifosfamide, Carboplatin, Etoposide), R-ICE (with rituximab), DHAP (Dexamethasone, Cytarabine, Cisplatin), R-DHAP (with rituximab), plus G-CSF. Two patients with lymphoma received cytostatics in monotherapy: one Endoxan and one AraC treatment. Aphereses were started when the number of CD34+ cells in peripheral blood was ≥10/µL. Flow cytometry counting of CD34+ cells was assessed. Apheresis was performed using a Spectra Optia device. In patients mobilized with chemotherapy and the granulocyte growth stimulation factor (G-CSF), the median length of G-CSF administration until the first apheresis was nine days (range: 5–22).

### 2.1. RNA Isolation and cDNA Synthesis

RNA was isolated using the miRNeasy Serum/Plasma KIT (Qiagen, Hilden, Germany) according to the manufacturer’s protocol. For qPCR miRNA analysis, TaqMan MicroRNA assays (Thermo Fisher Scientific, Waltham, MA, USA) were used with the following primers: hsa-miR-15a-5p, hsa-miR-16-5p, hsa-miR-34a-5p, hsa-miR-126-3p, hsa-miR-146a-5p, hsa-miR-155-5p, and hsa-miR-223-3p. Conversion of miRNA to cDNA was performed by incubation of 10 ng of total RNA with 3 µL of specific reverse transcription (RT) primer, 0.15 µL of 100 mMdNTPs, 1 µL of reverse transcriptase, 1.50 µL of 10x buffer, 0.19 µL of RNase inhibitor, and 4.16 µL of nuclease-free water in a 15-µL reaction (TaqMan MicroRNA Reverse Transcription Kit, Thermo Fisher Scientific). RT conditions were normalized using 5 nmol mirVana miRNA Mimic (cel-miR-39) as an exogenous control (Thermo Fisher Scientific, Waltham, MA, USA). The thermal condition steps of the reverse transcription reaction were as follows: 1–16 °C for 30 min; 2–42 °C for 30 min; 3–85 °C for 5 min; 4 °C for ∞.

### 2.2. qPCR miRNA Expression

Every qPCR reaction consisted of 4.5 µL of RT product diluted with nuclease-free water in a 1:15 ratio, 5 µL of TaqMan Fast Advanced Master Mix (Thermo Fisher Scientific, Waltham, MA, USA), and 0.5 µL of MicroRNA Assay. Samples were run in duplicate. An Applied Biosystems 7900HT Fast Real-Time PCR System machine was used for real-time PCR evaluation, according to the manufacturer’s recommendations. The following thermal conditions were set: initial activation at 95 °C for 10 min, 40 cycles of denaturation at 95 °C for 15 s, and annealing/extension at 60 °C for 60 s. SDS 2.4 and RQ Manager 1.2 software (Thermo Fisher Scientific) were used for the miRNA expression assay. Relative expression was calculated according to the Ct method 2^−∆∆CT^.

### 2.3. Statistical Analysis

The Wilcoxon signed-rank test was used to compare groups of dependent continuous variables: miRNA RQ (relative quantification) levels at two different time points. Spearman’s rank correlation coefficient was used to measure the statistical dependence between two variables. The Mann–Whitney U-test was used to compare independent variables: number of collected CD34+ cells during the first apheresis and miRNA RQ level; miRNA expression in good and poor mobilizers on day A. Statistical significance was set at *p* < 0.05.

## 3. Results

### 3.1. Mobilization Data

The median number of total collected CD34+ cells during mobilization was 5.07 × 10^6^/kg (range: 2.2–21). The median number of CD34+ cells collected after the first apheresis was 3 × 10^6^/kg (range: 0.3–21). The median number of CD34+ cells estimated in peripheral blood on the day of the first apheresis was 51.2/µL (range: 4.8–449.5). The median number of apheresis attempts needed to collect at least 2 × 10^6^/kg CD34+ was 2 (range: 1–6). Detailed clinical data of the patients enrolled in the study with miRNA raw data (RQ levels) are presented in Table S1.

### 3.2. miRNA Expression and Mobilization Efficacy

To assess the effectiveness of mobilization, the miRNA expression was tested against (1) the number of CD34+ cells in peripheral blood at the first apheresis, (2) the number of CD34+ cells collected on the day of the first apheresis, (3) the total number of CD34+ cells collected during mobilization, and (4) the number of apheresis attempts.

#### 3.2.1. miRNA Expression and the Number of CD34+ Cells in Peripheral Blood at First Apheresis

Negative correlations were observed between hsa-miR-15a-5p and hsa-miR-146a-5p expression on day A and the CD34+ number in the peripheral blood at the same time point (R = −0.30, *p* = 0.04), (R = −0.37, *p* = 0.008), respectively.

#### 3.2.2. miRNA Expression and the Number of Collected CD34+ Cells on the Day of First Apheresis

No correlation was observed between the expression of miRNAs on day 0 and the number of collected CD34+ cells at the first apheresis. Only for hsa-miR-146a-5p was a negative correlation observed between its expression on day A and the number of CD34+ cells collected at the first apheresis (R = −0.32, *p* = 0.02).

#### 3.2.3. miRNA Expression and the Total Number of CD34+ Cells Collected during Mobilization

Negative correlations were found between hsa-miR-15a-5p, hsa-miR-146a-5p, and hsa-miR-223-3p levels on day A and the total number of collected CD34+ cells (R = −0.30, *p* = 0.03), (R = −0.37, *p* = 0.007), (R = −0.30, *p* = 0.03), respectively.

**Figure 2:**
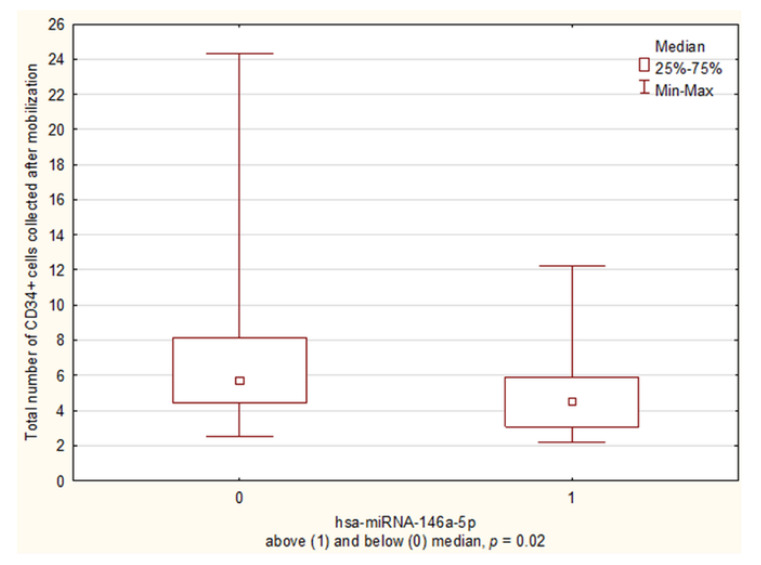
The comparison of the total number of CD34+ cells collected after mobilization at hsa-miR-146a-5p “low” and “high” expressors on the day of the first apheresis.

To evaluate the influence of miRNA expression on the total number of CD34+ cells collected after mobilization, patients were divided into “high” and “low” expression groups according to median miRNA levels on day A (above and below median). The group of hsa-miR-146a-5p “low expressors” collected more total CD34+ ×10^6^/kg cells than “high expressors” (5.67 vs. 4.33 CD34+ ×10^6^/kg, *p* = 0.02) (Figure 2).

#### 3.2.4. miRNA Expression and the Number of Apheresis Attempts

Positive correlations were noticed between hsa-miR-15a-5p, hsa-miR-126-3p, and hsa-miR-146a-5p expressions on day A and the number of apheresis attempts (R = 0.34, *p* = 0.02), (R = 0.29, *p* = 0.04), (R = 0.35, *p* = 0.01).

### 3.3. miRNA Expression in Good and Poor Mobilizers According GITMO Criteria

For better evaluation of miRNA expression’s influence on mobilization efficacy, patients were divided into “poor” (*n* = 6) and “good” (*n* = 44) mobilizer groups. The “poor mobilizer” group was defined according to the Italian Group for Stem Cell Transplantation (GITMO) criteria [62]: lymphoma/myeloma patients in whom, after adequate mobilization (G-CSF 10 µg/kg if used alone or ≥5 µg/kg after chemotherapy), the circulating CD34+ cell peak was <20/µL up to 6 days after mobilization with G-CSF, or up to 20 days after chemotherapy and G-CSF, or if they yielded <2 × 10^6^ CD34+ cells per kg in ≤3 aphereses.

**Figure 3:**
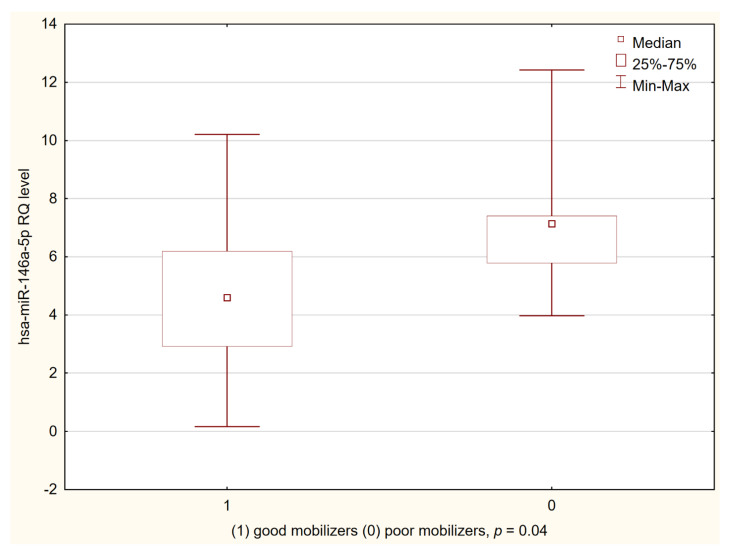
hsa-miR-146a-5p expression on the day of the first apheresis in patients divided into “good” and “poor” mobilizers according to GITMO criteria.

The “good mobilizers” had a lower hsa-miR-146a-5p level on the day of the first apheresis than the “poor mobilizers” (Me = 4.56 vs. 7.11, *p* = 0.04) (Figure 3). No significant differences were found in the expression of other miRNAs in either the “poor” or the “good” mobilizer groups.

#### miRNA Expression According to CD34 Peak in Peripheral Blood

A negative correlation was also found between hsa-miR-146a-5p expression and the total CD34+ cell count collected in the good mobilizer group (R = −0.38, *p* = 0.006). A multivariate analysis was performed to identify factors influencing the achievement of ≥20 CD34+ cells/µL in peripheral blood before the first apheresis. Clinical parameters (sex, age, quality of myeloma/lymphoma response) and the levels of hsa-miR-15a-5p and hsa-miR-146a-5p at the day of apheresis were taken into account. The only factor associated with an adequate CD34+ peak in peripheral blood in univariate analysis was hsa-miR-146a-5p expression on day A, with an odds ratio of 1.88 (95% CI, 1.06–3.33, *p* = 0.03) (Table 2). According to multivariate regression, hsa-miR-146a-5p level on day A was an independent factor for mobilization of a sufficient number of CD34+ cells (2 × 10^6^/kg).

### 3.4. Kinetics of miRNA

**Figure 4:**
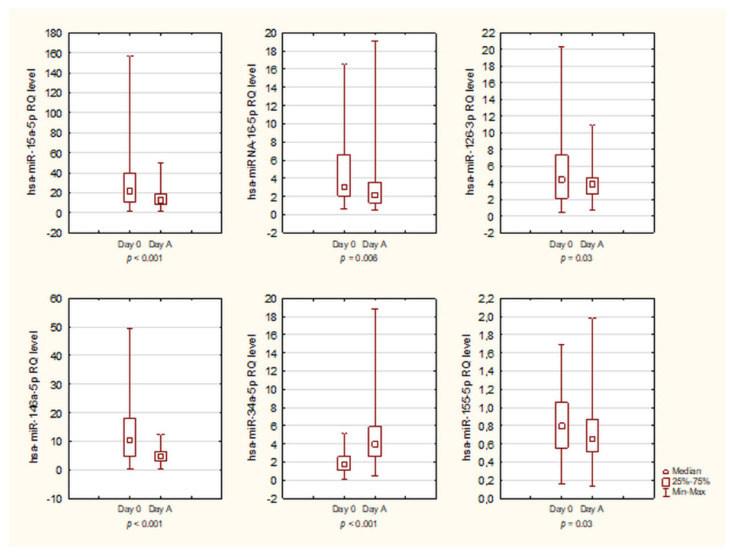
The kinetics of miRNAs expression at two time points: the day before HSC mobilization (day 0) and the day of the first apheresis (day A): hsa-miR-15a-5p, hsa-miR-16-5p, hsa-miR-126-3p, hsa-miR-146a-5p, hsa-miR-34a-5p, and hsa-miR-155-5p.

The level of miRNA expression was evaluated at two time points: before the mobilization of HSC (day 0) and on the day of the first apheresis (day A). Statistical analysis was performed using the Wilcoxon matched-pairs test. Our study revealed a profound decrease the hsa-miR-15a-5p, hsa-miR-16-5p, hsa-miR-126-3p, hsa-miR-146a-5p, and hsa-miR-155-5p expression on day A compared to day 0 (baseline). hsa-miR-34a-5p expression was increased after mobilization chemotherapy compared to day 0 (Figure 4). No significant change in hsa-miR-223-3p expression was observed between day 0 and day A. The levels of investigated miRNAs during the mobilization period are presented in Table 3.

To evaluate the influence of miRNA expression on the total number of CD34+ cells collected after mobilization, the number of CD34+ cells collected on Day A, and the CD34+ peak in peripheral blood on Day A, patients were divided into “increase” and “decrease” expression groups. The “increase” group consisted of patients with an observed increase in the expression of each miRNA on Day A compared to Day 0. The “decrease” group consisted of patients with an observed decrease in expression of each miRNA on Day A compared to Day 0. The number of patients from “increase” and “decrease” groups was as follows: hsa-miR-15a-5p (*n* = 14 vs. *n* = 36), hsa-miR-16-5p (*n* = 13 vs. *n* = 37), hsa-miR-126-3p (*n* = 20 vs. *n* = 30), hsa-miR-146a-5p (*n* = 10 vs. *n* = 40), and hsa-miR-155-5p (*n* = 18 vs. *n* = 32). We found no statistically significant differences between the “increase” and “decrease” groups in relation to the analyzed mobilization parameters. Results of the analysis are presented in Supplementary Table S2.

### 3.5. Relationship between WBC Count and miRNA Expression

The correlation between miRNA expression and granulopoiesis during the mobilization procedure was determined using the Spearman’s rank correlation coefficient analysis. White blood cell (WBC) count was assessed on the day of the first apheresis. A positive correlation was observed only between hsa-miR-223-3p level and WBC (R = 0.39, *p* = 0.005).

### 3.6. miRNA Expression and Remission Status

The relationship between both miRNA expression on day 0 and day A and the depth of myeloma/lymphoma response (CR vs. not CR before mobilization chemotherapy) was also tested. Of these, only hsa-miR-146a-5p demonstrated a negative correlation between the expression on day 0 and the depth of response after previous treatment (R = −0.33, *p* = 0.02).

## 4. Discussion

MicroRNAs are molecules involved in the differentiation and migration of the HSC in the bone marrow niche [6,7]. In our previous studies, we found that miRNAs might affect the migration of HSC in the post-transplantation period, and changes in their expression are associated with the efficiency of regeneration after auto-HSCT [25]. However, although microRNAs influence CD34+ cell migration from the bone marrow niche to peripheral blood, their impact on the efficiency of mobilization remains unknown. In our study, we assessed selected miRNAs at the key time points of this procedure.

In the present study, the expressions of hsa-miR-15a-5p, hsa-miR-16-5p, hsa-miR-126-3p, hsa-miR-146a-5p, and hsa-miR-155-5p significantly decreased during the process of CD34+ cell mobilization compared to the baseline. In contrast, only hsa-miR-34a-5p expression was upregulated. Chemotherapy impairs the proliferation and migration of hematopoietic cells, which can promote a significant decrease in miRNA expression [63,64]. A similar relationship was observed in patients after the administration of conditioning chemotherapy [25].

In our study, a slight decrease in hsa-miR-126-3p expression on the day of the first apheresis, as compared to the baseline, was observed. It is likely that mobilization chemotherapy inhibited its expression. However, microvesicles containing hsa-miR-126-3p have been found to occur during mobilization, and they are associated with the suppression of VCAM1 expression [15,38]. This, in turn, allows an easier release of HSC from the bone marrow niche to peripheral blood, especially under G-CSF stimulation [15,36,38].

For hsa-miR-34a-5p, a significant increase in the expression was noted. It is likely that this miRNA also deregulates directly after mobilization chemotherapy. We suppose that the stimulation of G-CSF appears to significantly increase hsa-miR-34a-5p expression until apheresis begins. Increased hsa-miR-34a-5p expression affects the NOTCH1, AKT1, and MAPK1/MAPK3 signaling pathways, which may be involved in HSC migration from the bone marrow niche to peripheral blood [21,35].

Surprisingly, on the day of the first apheresis, hsa-miR-223-3p levels did not significantly differ from the baseline. Hsa-miR-223-3p is a key molecule that regulates granulopoiesis [56,57,58] and, as such, it was surprising that no such change happened after G-CSF stimulation [23]. It is worth noting, however, that an upward trend in hsa-miR-223-3p expression was observed. After mobilization chemotherapy, a decrease in WBC is observed and the processes of regeneration of the marrow niche, including granulopoiesis, follow. In our previous studies, we noted a significant decrease in the hsa-miR-223-3p expression after conditioning chemotherapy [25]. We suppose that a relatively stable level of hsa-miR-223-3p observed in our study might be a result of a G-CSF co-stimulation, which significantly affects granulopoiesis and presumably also hsa-miR-223-3p concentration. On the day of the first apheresis, the patients had a relatively high level of WBC following several days of G-CSF stimulation, and the granulopoiesis process was not as intense as at the end of chemotherapy. Hence, lower hsa-miR-223-3p levels result in a higher number of CD34+ cells after the first apheresis. In addition, a positive correlation was observed between hsa-miR-223-3p expression and the total WBC count. Our findings are in line with previous studies that indicate that hsa-miR-223-3p expression significantly affects granulopoiesis, which accelerates during G-CSF stimulation [55,57]. This observation confirms findings from our previous studies, where a positive correlation was found between WBC count and hsa-miR-223-3p level after auto-HSCT [25].

Regarding the effect on the depth of response after the previous chemotherapy and miRNA expression before mobilization chemotherapy, patients in CR demonstrated a lower hsa-miR-146a-5p expression. In previous studies, hsa-miR-146a-5p was considered a tumor suppressor, and its reduced expression was associated with the growth of many hematological cancers [40,41,42]. This miRNA, by affecting the NFKB1 pathway, participates in the inflammatory response, and also affects the development of myeloid and lymphoid tumors [40,41,42]. We suppose that, in complete remission, these signaling pathways are not as severely disturbed as they are during progression, where the increased expression of hsa-miR-146a-5p is observed [65].

Alterations in hsa-miR-146a-5p expression may be important for the hematopoiesis and effective mobilization of CD34+ cells [43,44]. Hsa-miR-146a-5p expression influenced the number of CD34+ cells in peripheral blood on the day of the first apheresis, as well as the amount of harvested CD34+ cells after the first apheresis. In the context of G-CSF stimulation, hsa-miR-146a-5p significantly affects the CXCL12/CXCR4 signaling pathway, which is associated with HSC migration [43]. Hsa-miR-146a-5p influences CXCR4 mRNA expression, which results in the disruption of the CXCL12/CXCR4 complex and subsequent release of CD34+ HSC [20,43,66]. Previous findings indicating the regulatory role of hsa-miR-146a-5p in the CXCL12/CXCR4 axis were performed in vitro in different cell lines [20,43] Our study is the first one evaluating the hsa-miR-146a-5p level in sequential patients’ PB samples and correlating its expression with CD34+ cells number in peripheral blood. In our previous research, low hsa-miR-146a-5p expression was observed after auto-HSCT, in the nadir of bone marrow aplasia; this facilitated HSC homing and efficient adhesion in the hematopoietic niche [25].

Interestingly, upregulated hsa-miR-146a-5p expression accompanied by reduced cell proliferation and increased sensitivity to chemotherapy was observed in leukemia cells [67]. Decreased hsa-miR-146a-5p expression was also seen in these cells during monocyte differentiation [43,67]. The downregulated expression of hsa-miR-146a-5p is associated with increased hematopoietic differentiation, including megakaryocytes [43,67]. Since the G-CSF stimulates these processes in the bone marrow, low levels of hsa-miR-146a-5p do not negatively affect the effectiveness of mobilization. It is probable that the impact of this miRNA on the CXCL12/CXCR4 signaling pathway is only one of many factors affecting HSC migration.

Patients who were defined as good mobilizers according to the GITMO criteria had lower hsa-miR-146a-5p expression than poor mobilizers. Furthermore, patients who had lower hsa-miR-146a-5p expression on the day of the first apheresis obtained more CD34+ cells during apheresis. These results might suggest that low hsa-miR-146a-5p has a more relevant impact on hematopoietic precursors than on the CXCL12/CXCR4 pathway, and does not disturb CD34+ cells’ migration from the hematopoietic niche to peripheral blood. We assume that the reduced expression of hsa-miR-146a-5p positively affects the mobilization of CD34+ cells.

Expression of hsa-miR-15a-5p was lower in patients who had a high CD34+ cell number in peripheral blood. These patients also obtained more CD34+ cells after the first apheresis. The hsa-miR-15a-5p/-16-5p cluster expression inversely correlates with VEGFA expression and significantly influences hematopoiesis and bone marrow reconstitution after HSCT [13,29]. This process is particularly evident in myeloma cells [13]. Moreover, downregulation of the hsa-miR-15a-5p/-16-5p cluster influences AKT1 signaling and regulates HSC migration. A low level of hsa-miR-15a-5p/-16-5p influences VEGFA expression, which promotes proliferation and HSC mobilization and inhibits apoptosis [13,30,68].

We are aware that the number of patients was certainly a limiting factor in our research. However, we would like to point out that our results indicate the direction of further research on the effectiveness of mobilization in a larger group of patients. Hsa-miR-146a-5p may be an important prognostic factor for the effectiveness of CD34+ cell mobilization. The other limitation of our study is the heterogeneity of the population due to the inclusion of all consecutive patients with lymphoproliferative disorders scheduled for auto-HSCT in our department. The number of lymphoma patients, however, is too small to evaluate lymphoma and myeloma patients separately. Moreover, when analyzing the kinetics of miRNA expression, although the miRNAs expression medians showed an overall decline after mobilization, it was observed that, in some patients, there was an increase in more than one miRNA from day 0 to day A. Exploring the expression of miRNAs in larger cohorts seems warranted, and this should be conducted separately for myeloma and lymphoma patients and for different chemotherapy regimens.

In conclusion, miRNAs play an important role in the migration of HSC and can affect the efficiency of CD34+ cell mobilization for future auto-HSCT. miRNAs may be predictive biomarkers for a successful mobilization of CD34+ cells. The exact role of miRNAs active in the hematopoietic niche, especially hsa-miR-146a-5p in the process of mobilization, is worth exploring.

## Figures and Tables

**Table 1:** Characteristics of the patients enrolled to the study.

Characteristics	Numbers
Age (years)	Median 60 (range 44–69)
Sex (female/male)	25/25
Multiple myeloma	39 (7 CR, 24 VGPR, 8 PR)
Hodgkin lymphoma	4 (1 CR, 3 PR)
non-Hodgkin lymphoma:	7
Diffuse large B-cell lymphoma	3 (1 CR, 2 PR)
Mantle cell lymphoma	2 (CR)
Anaplastic large-cell lymphoma	1 (PR)
Hepatosplenic T-cell lymphoma	1 (PR)
CD34+ cells collected during mobilization (total number) [×10^6^/kg]	Median 5.07 (range 2.2–21)
CD34+ collected on Day A [×10^6^/kg]	Median 3.0 (range 0.3–21)
Number of apheresis needed to collect at least 2 × 10^6^/kg CD34+	Median 2 (range 1–6)
WBC count on Day A [×10^3^/µL]	Median 16.67 (range 2.68–47.42)
Mobilization chemotherapy:	
Multiple myeloma	
Endoxan (Cyclophosphamide)	25
DCEP (Dexamethasone, Cyclophosphamide,	7
Cisplatin, Etoposide)	
Alexan (Cytarabine)	3
only G-CSF in monotherapy	5
Hodgkin and non-Hodgkin lymphoma	
ICE (Ifosfamide, Carboplatin, Etoposide)	4
R-ICE (with rituximab)	1
DHAP (Dexamethasone, Cytarabine, Cisplatin)	1
R-DHAP (with rituximab)	2
Endoxan (Cyclophosphamide)	1
Alexan (Cytarabine)	1
Mobilization efficacy	
Good mobilizers	44
Poor mobilizers	6

Best response achieved prior to mobilization procedure: CR—complete remission, VGPR—very good partial remission (only multiple myeloma), PR—partial remission.

**Table 2:** Univariate analysis of factors associated with achievement of at least 20 CD34+ cells/µL in peripheral blood before first apheresis.

Factor	Odds Ratio (95% CI)	*p* Value
Male vs. female	1.64 (0.26–10.21)	0.59
Age (continuous)	1.03 (0.94–1.13)	0.46
CR vs. not CR	1.17 (0.13–10.89)	0.89
hsa-miR-15a-5p (Day 0)	1.01 (0.96–1.07)	0.69
hsa-miR-146a-5p (Day 0)	0.99 (0.86–1.13)	0.89
hsa-miR-15a-5p (Day A)	0.89 (0.74–1.08)	0.23
hsa-miR-146a-5p (Day A)	1.88 (1.06–3.33)	0.03

**Table 3:** Changes in miRNA expression (RQ) before mobilization chemotherapy and on the day of the first apheresis.

miRNA	Day 0	Day A	*p* Value
hsa-miR-15a-5p	Me = 21.92 range: 1–156.83	Me = 12.67 range: 1.24–50.35	*p* < 0.001
hsa-miR-16-5p	Me = 3.03 range: 0.64–16.53	Me = 2.14 range: 0.52–19.07	*p* = 0.006
hsa-miR-126-3p	Me = 4.39 range: 0.46–20.38	Me = 3.83 range: 0.69–10.9	*p* = 0.03
hsa-miR-146a-5p	Me = 10.42 range: 0.40–49.52	Me = 4.74 range: 0.16–12.42	*p* < 0.001
hsa-miR-223-3p	Me = 14.51 range: 0.69–105.31	Me = 15.69 range: 1.9–55.84	*p* = 0.66
hsa-miR-34a-5p	Me = 1.74 range: 0.04–5.13	Me = 3.95 range: 0.48–18.78	*p* < 0.001
hsa-miR-155-5p	Me = 0.80 range: 0.16–1.69	Me = 0.66 range: 0.13–1.98	*p* = 0.03

Day 0—the day before hematopoietic stem cell mobilization, day A—the day of the first apheresis.

## Data Availability

Not applicable.

## Supplementary Materials

The following materials are available online at https://www.mdpi.com/article/10.3390/biology10070668/s1, Table S1: Clinical characteristics of the patients enrolled in the study along with the RQ level of miRNAs determined in patients on day 0 and day A. Table S2: The comparison of the total number of CD34+ cells collected after mobilization, number of cells collected at first apheresis, and CD34+ peak in peripheral blood at miRNAs “increase” and “decrease” expression groups from Day 0 to day A.

## Abbreviations

HSC	Hematopoietic Stem Cells
HSCT	Hematopoietic Stem Cell Transplantation
LD	Lymphoproliferative Disorders
G-CSF	Granulocyte Colony-stimulating Factor
EC	Endothelial Cells
HPSC	Hematopoietic Progenitor and Stem Cells
PB	Peripheral Blood
