# Supplementary material for: Alterations in microRNA Expression during Hematopoietic Stem Cell Mobilization
Source: Biology (Basel). 2021 Jul 15;10(7):668. doi: 10.3390/biology10070668 (PMC8301406; doi:10.3390/biology10070668)
Supplement: Supplementary file 1 [file biology-10-00668-s001.zip › biology-1261025-supplementary.pdf]

Table S1. Clinical characteristics of the patients enrolled in the study along with the RQ level of miRNAs determined in patients on day 0 and day A.

Patient number	Sex	Age	Diagnosis	Response 1 –CR, 0 – not CR	Number of apheresis	Number of CD34+ cells on day A	CD34+/µl peak in peripheral blood on day A	Total number of collected CD34+ cells	WBC [$\times 10^3$/µl] in peripheral blood at day A	RQ level on Day 0							RQ level on Day A						
										miRNA-15a	miRNA-16	miRNA-126	miRNA-146a	miRNA-223	miRNA-34a	miRNA-155	miRNA-15a	miRNA-16	miRNA-126	miRNA-146a	miRNA-223	miRNA-34a	miRNA-155
1	M	62	MM	CR	1	7.1	115.4	7.1	20.39	4.817	1.371	1.233	4.024	6.231	1.053	1.438	3.688	2.308	1.832	1.357	7.667	4.585	1.141
2	F	57	MM	CR	3	0.9	12.3	3.05	33.36	4.925	0.881	1.491	2.115	1.949	1.433	0.909	18.34	2.472	4.028	8.021	32.414	0.49	0.131
3	M	65	MM	CR	1	4.97	103.2	4.97	23.3	37.487	2.036	5.436	9.147	12.945	2.286	1.344	14.483	1.621	3.986	5.976	15.933	5.868	0.343
4	M	63	MM	CR	4	0.32	5.5	2.5	27.31	4.501	0.64	1.165	1.518	1.131	0.654	0.508	12.548	1.262	3.855	3.978	11.661	2.239	0.457
5	M	48	MM	CR	1	7.27	94	7.27	9.4	15.576	2.086	3.241	6.031	8.882	0.986	0.539	4.688	1.497	1.126	1.061	3.195	2.615	0.891
6	F	51	MM	CR	1	4.25	56.8	4.25	17.64	39.807	8.742	7.489	13.156	25.455	3.068	1.47	13.51	3.349	3.948	2.793	15.438	2.917	1.271
7	M	60	MM	CR	1	10.8	88.8	10.8	14.85	15.517	5.204	2.057	3.774	6.511	0.662	1.201	18.381	2.829	5.561	7.673	19.644	3.267	0.814
8	F	61	MM	not CR (VGPR)	2	3.2	58.4	5.58	17.04	3.166	2.791	1.176	0.862	1.34	1.26	1.687	9.076	5.205	7.258	4.265	18.165	5.552	1.787
9	F	65	MM	not CR (VGPR)	2	1.5	25.3	2.91	13.44	38.442	10.066	6.072	12.672	27.48	1.909	1.195	29.216	9.259	5.751	9.432	55.839	2.777	0.664
10	M	67	MM	not CR (VGPR)	6	0.82	7.4	2.99	30.85	6.772	2.126	0.94	3.531	3.689	0.042	0.466	21.168	2.435	4.849	12.421	28.394	3.251	0.977

11	F	54	MM	not CR (VGPR)	2	1.7	24.2	24.3	3.16	43.348	7.133	8.071	19.123	29.673	4.774	0.988	12.387	4.26	4.287	4.012	8.658	6.106	0.87
12	F	39	MM	not CR (VGPR)	2	2.7	26.5	5.88	7.71	41.216	11.596	7.975	13.775	36.867	3.153	1.35	14.411	7.388	3.153	4.567	17.45	16.211	0.97
13	F	51	MM	not CR (VGPR)	1	4.52	132.2	4.52	26.28	6.197	5.856	0.917	0.878	1.272	2.564	1.053	14.354	1.938	2.853	5.327	19.393	5.821	0.767
14	F	57	MM	not CR (VGPR)	1	4.77	44.3	4.77	14.48	1.845	1.303	0.636	0.943	0.895	1.463	0.632	8.247	1.444	1.68	4.965	16.564	5.171	0.416
15	M	65	MM	not CR (VGPR)	2	4.59	59.3	11.58	9.83	3.4	2.424	0.457	0.399	0.69	1.139	0.392	11.182	4.349	1.872	2.711	7.578	4.312	0.523
16	F	62	MM	not CR (VGPR)	1	8.7	109.7	8.7	26.63	77.243	16.525	10.542	36.965	77.805	5.13	0.95	10.731	7.096	2.835	4.26	12.619	3.914	0.713
17	F	69	MM	not CR (VGPR)	1	6.1	91.2	6.1	20.78	37.867	3.233	5.99	18.141	24.861	1.21	0.423	5.956	6.366	4.009	5.2	18.838	4.044	0.309
18	F	42	MM	not CR (VGPR)	3	1.15	11.34	4.5	6.75	48.638	2.346	7.307	18.301	23.236	1.766	1.236	9.775	2.101	3.802	6.171	12.732	2.952	0.615
19	M	63	MM	not CR (VGPR)	1	13.5	179.3	13.5	17.19	18.819	1.449	3.672	7.021	13.142	1.395	1.012	13.485	1.307	4.334	4.65	16.522	6.26	0.565
20	M	50	MM	not CR (VGPR)	2	2.49	89.9	5.29	18.82	22.114	9.339	3.087	4.919	8.039	1.959	0.262	8.632	1.561	3.073	2.801	13.056	5.704	0.462
21	F	51	MM	not CR (VGPR)	4	0.3	5.1	2.66	21.99	8.741	2.991	2.745	12.393	8.034	0.535	0.514	10.79	0.941	2.871	5.793	10.312	0.483	0.635
22	M	60	MM	not CR (VGPR)	1	3.5	51.9	3.5	16.56	68.182	7.078	14.28	47.676	61.797	4.838	0.713	9.955	2.125	2.999	4.631	12.515	2.945	0.695
23	M	60	MM	not CR (VGPR)	1	20.8	415.4	20.8	14.45	21.62	2.634	9.606	30.81	43.928	1.06	0.249	11.841	1.474	3.322	4.01	12.638	2.813	0.613
24	M	55	MM	not CR (VGPR)	1	5.69	143.5	5.69	10.93	42.359	4.212	8.132	19.106	25.865	1.257	0.971	1.237	0.519	2.168	3.7	4.047	0.814	0.439
25	M	50	MM	not CR (VGPR)	1	21	449.5	21	9.79	53.106	3.48	10.149	40.938	46.566	1.611	0.597	6.036	1.079	1.777	2.456	4.858	2.326	0.7

26	F	63	MM	not CR (VGPR)	4	0.6	4.8	2.2	14.35	11.258	2.22	2.312	8.66	4.762	2.719	0.848	16.968	2.465	5.073	7.167	16.866	3.984	0.926
27	F	62	MM	not CR (VGPR)	4	0.78	11.9	3.1	15.65	31.242	4.399	5.583	10.002	15.391	2.121	1.267	20.879	3.554	5.338	7.4	23.073	3.288	0.792
28	F	60	MM	not CR (VGPR)	3	0.46	7.59	3.6	22.33	22.545	2.482	5.005	14.43	22.044	1.061	0.734	22.052	2.147	6.202	7.975	31.786	2.104	0.637
29	F	65	MM	not CR (VGPR)	5	0.49	6.9	2.72	7.34	156.829	10.713	20.38	46.847	105.312	4.966	0.981	23.304	6.582	4.416	7.105	18.786	7.985	1.982
30	F	62	MM	not CR (VGPR)	1	7.5	130.4	7.5	47.42	1	1	1	1	1	1	1	2.387	0.648	1.761	1.122	9.479	2.045	1.258
31	F	60	MM	not CR (VGPR)	1	5.3	71.5	5.3	19.44	25.037	6.606	5.686	10.132	14.393	2.445	1.285	16.928	2.133	5.491	6.204	23.183	6.068	0.821
32	F	55	MM	not CR (PR)	2	0.9	14.3	3.42	4.13	23.915	3.676	2.89	12.752	14.619	0.844	0.708	15.303	6.385	4.007	10.201	21.545	18.778	0.472
33	M	40	MM	not CR (PR)	1	4.7	62.6	4.7	12.6	16.646	7.874	4.003	4.805	13.367	2.128	1.127	18.536	16.312	4.579	3.592	25.085	13.391	0.647
34	M	56	MM	not CR (PR)	2	2.62	27.6	4.46	28.77	49.926	11.175	7.516	13.743	38.17	2.591	0.791	33.237	19.073	5.791	6.213	33.385	4.82	1.017
35	M	60	MM	not CR (PR)	1	6.47	153.6	6.47	2.68	22.408	2.078	5.712	15.418	17.117	1.934	0.636	10.232	1.993	2.708	5.066	9.723	4.868	0.67
36	M	44	MM	not CR (PR)	2	1.79	16.6	3.02	4.69	40.146	3.107	6.506	19.159	16.49	1.752	0.913	12.79	1.128	3.998	5.668	9.148	3.03	0.79
37	M	63	MM	not CR (PR)	1	5.8	95.6	5.8	3.4	9.302	1.839	2.165	9.191	11.156	1.734	0.156	1.923	0.603	0.688	1.485	1.897	2.551	0.242
38	M	64	MM	not CR (PR)	2	1.2	18	8.15	8.76	27.533	2.823	5.578	19.567	19.17	2.185	0.676	6.56	1.295	1.779	0.163	6.005	3.396	0.518
39	M	36	MM	not CR (PR)	1	8.2	84.2	8.2	6.16	77.573	5.759	17.696	49.592	58.687	1.676	0.866	21.031	2.24	4.603	8.035	16.443	3.604	0.369
40	M	54	NHL	CR	3	1.14	24.5	3.36	21.89	18.593	5.053	3.469	4.895	11.515	1.34	0.556	7.062	1.352	2.059	2.111	11.229	5.583	0.643

			(MCL)																				
41	F	60	NHL (MCL)	CR	2	6.7	156.9	10.6	30.34	33.839	12.738	5.945	10.713	34.125	3.383	1.257	17.556	3.443	3.787	4.828	25.496	7.083	1.126
42	M	53	NHL (DLBCL)	CR	1	3.9	79.4	3.9	14.34	9.533	1.778	2.056	8.17	6.046	1.051	0.643	8.312	1.215	3.192	3.313	10.309	4.24	0.522
43	F	62	NHL (DLBCL)	not CR (PR)	2	2.6	46.5	4.42	37.85	15.784	1.536	3.401	12.105	13.714	3.024	0.738	24.142	2.78	4.569	3.853	37.098	7.556	0.643
44	M	59	NHL (DLBCL)	not CR (PR)	2	1.68	27.18	4.98	16.78	21.717	1.783	6.088	13.227	15.486	2.696	0.622	13.379	1.174	2.681	5.764	9.983	2.367	0.324
45	F	42	NHL (HSTL)	not CR (PR)	3	1.85	21.1	3.59	40.5	14.781	1.622	2.475	6.088	9.901	0.949	0.323	25.583	2.65	8.15	8.539	25.348	8.586	0.824
46	F	60	NHL (ALCL)	not CR (PR)	2	1.65	29.6	5.43	11.25	10.582	0.7	1.678	4.612	6.134	0.612	0.182	5.292	0.903	1.611	3.045	4.729	0.972	0.585
47	F	38	HL	CR	2	2.8	29.5	4.33	29.8	19.13	3.742	4.073	8.283	14.841	1.795	0.751	12.111	1.366	2.926	3.172	14.304	1.48	0.354
48	M	49	HL	not CR (PR)	2	2.26	22.5	5.16	33.16	12.914	3.06	2.606	3.555	8.866	1.496	0.908	6.648	0.68	1.958	1.89	4.012	1.812	0.934
49	F	34	HL	not CR (PR)	2	2.3	50.5	5.9	8.08	29.767	2.342	4.707	15.221	24.319	2.592	0.501	27.948	3.574	5.329	9.26	24.412	12.376	0.679

50	M	21	HL	not CR (PR)	1	12.2	205.5	12.2	42.73	119.217	8.908	16.958	31.423	79.905	3.762	0.804	50.351	6.079	10.896	6.551	42.984	7.977	0.531

Table S2. The comparison of the total number of CD34+ cells collected after mobilization. number of cells collected at first apheresis. and CD34+ peak in peripheral blood at miRNAs "increase" and "decrease" expression groups from Day 0 to day A**.**

miRNA	Total number of CD34+ cells collected after mobilization [x10⁶/kg]			Number of collected CD34+ on Day A [x10⁶/kg]			Number of CD34+ cells in peripheral blood on Day A [cells/μL]		
	Increase group	Decrease group	p	Increase group	Decrease group	p	Increase group	Decrease group	p
hsa-miR-15a-5p	Me = 4.41	Me = 5.22	P = 0.08	Me = 2.88	M = 3.11	P = 0.26	Me = 43.55	Me = 52.11	P = 0.13
hsa-miR-16-5p	Me = 4.73	M = 5.10	P = 0.38	Me = 2.30	M = 3.86	P = 0.17	Me = 43.55	Me = 62.25	P = 0.17
hsa-miR-126-3p	Me = 4.48	Me = 5.29	P = 0.13	Me = 2.88	Me = 3.11	P = 0.27	Me = 46.37	Me = 53.58	P = 0.22
hsa-miR-146a-5p	Me = 4.60	Me = 5.16	P = 0.57	Me = 3.85	Me = 2.76	P = 0.80	Me = 50.76	Me = 52.11	P = 0.54
hsa-miR-155-5p	Me = 5.41	Me = 4.66	P = 0.60	Me = 2.48	Me = 3.68	P = 0.99	Me = 55.04	Me = 49.30	P = 0.79
